# Pressure ulcer is associated with malnutrition as assessed by Nutritional Risk Screening (NRS 2002) in a mixed hospital population

**DOI:** 10.1080/16546628.2017.1324230

**Published:** 2017-05-12

**Authors:** Johanne Alhaug, Caryl L Gay, Christine Henriksen, Anners Lerdal

**Affiliations:** ^a^ Department of Clinical Nutrition, Lovisenberg Diaconal Hospital, Oslo, Norway; ^b^ Department of Nutrition, University of Oslo, Oslo, Norway; ^c^ Department of Family Health Care Nursing, University of California, San Francisco, CA, USA; ^d^ Department of Research and Development, Lovisenberg Diaconal Hospital, Oslo, Norway; ^e^ Department of Research, Lovisenberg Diaconal Hospital, Oslo, Norway; ^f^ Institute of Nursing Science, Department of Health and Society, Faculty of Medicine, University of Oslo, Oslo, Norway

**Keywords:** Pressure ulcer, nutritional risk, NRS 2002, malnutrition, European Pressure Ulcer Advisory Panel (EPUAP)

## Abstract

**Background and aim**: Pressure ulcers (PUs) and malnutrition represent a significant health problem for hospital inpatients. Satisfactory nutritional status is crucial for proper wound healing. Risk of malnutrition can be identified using standardized screening tools, such as the Nutritional Risk Screening (NRS) 2002.

**Objective**: The objective of this study was to examine whether nutritional status based on the NRS 2002 is associated with PU in hospital inpatients.

**Design**: The data for this cross-sectional analysis were based on 10 screening days between September 2012 and May 2014. All adult inpatients admitted to a medical or surgical ward on the screening days were evaluated for eligibility. Nursing students and ward nurses conducted the NRS 2002 initial screening and skin examinations for PU classification (Stages I–IV). A registered clinical dietician conducted all NRS 2002 final screenings.

**Results**: The sample consisted of 651 patients, with mean age 62.9 years. Skin examinations indicated an 8% PU prevalence. Factors associated with PUs included age ≥ 70 years, low body mass index (BMI) and hospitalization in the medical department. Based on the initial screening, 48% were at ‘Low risk’ for malnutrition and 52% were at ‘Possible risk’. After final screening, 34% of the sample was identified as ‘At risk’ for malnutrition. Patients identified at ‘Possible risk’ by the initial screening or ‘At risk’ by the final screening were more likely than patients at ‘Low risk’ to have a PU (OR = 2.58 and 2.55, respectively). Each of the three initial screening items was significantly associated PU, with ‘Is BMI<20?’ and ‘Ate less past week?’ having the strongest associations.

**Conclusion**: Nutritional risk using the NRS 2002 is associated with the presence of PU in a mixed hospital population. The final screening had a slightly stronger association with PU compared to the initial screening.

## Introduction

Pressure ulcers (PUs) represent a significant health problem for patients admitted to hospitals [1–5]. The condition causes pain, decreases quality of life, increases risk of infection and morbidity, and leads to longer hospital stays [1,6,7]. The European Pressure Ulcer Advisory Panel (EPUAP) reported a PU prevalence of 18% among 6000 patients in a hospital setting based on data from five European countries [8]. Data from the United Kingdom, United States and Canada found PU prevalence between 5 and 32%, while Japan and China report 1–3% prevalence in hospital populations [6,9]. Recent Norwegian data indicate an 18% PU prevalence and 14% rate of hospital acquired pressure ulcers (HAPUs), with the highest prevalence in the intensive care units [10,11]. It is suggested that the risk of HAPUs decreases when patient safety routines and PU prevention guidelines are implemented [10]. Common risk factors for PUs include immobility, friction and shear, moisture, incontinence, poor nutrition, perfusion, older age, skin condition and altered level of consciousness [1,5,12,13].

Comparing PU prevalence data from different countries can be challenging, partly due to different patient populations and use of differing PU assessment methods [8]. Nevertheless, the importance of addressing PU risk and assessment as early as possible, to prevent and minimize PU development during hospitalization, is emphasized [5]. PU treatment is both invasive and costly, which has considerable impact on national healthcare budgets [14–16]. In the UK, the calculated cost is estimated to GBP 1.064 for Stage I to GBP 10.551 for Stage IV, with a total national expense of GBP 1.2–1.4 billion annually [14]. The Norwegian annual national healthcare cost for PU treatment has been estimated at NOK 700 million annually, 1% of the healthcare budget (GBP 52 million −2015 value) [17].

Guidelines, recommendations and research emphasize the significance of poor nutritional status for development of PUs [1,5,6,10,18]. Malnutrition is recognized as one of the major systemic risk factors for poor wound healing and developing PUs [3,18]. European estimates of malnutrition indicate a prevalence of 20–50% among hospital patients [19–21]. Malnutrition is associated with reduced immune response, poor wound healing, decreased physical and mental function, and increased length of hospital stay, morbidity and mortality [18,20,22–27].

Nutritional risk screening is a rapid and efficient method for detecting patients at risk of malnutrition. National guidelines recommend nutritional risk screening within 24 hours for all patients admitted to the national healthcare services, using validated screening tools [19,20]. The Norwegian Patients Safety Program (2014) recommends PU risk screening within 8 hours after hospital admission [28]. Early risk screening for PUs and malnutrition represent valuable routines for detecting patients at risk. However, risk screening can be time- and resource-consuming, which unfortunately often results in it being downgraded in regular routines [6,11,14]. Nevertheless, targeted identification of patients at nutritional risk may be useful in addressing risk of development and presence of PUs [1]. The objective of this study was to examine whether hospital inpatients at risk of malnutrition according to the NRS 2002 screening tool are more likely to have PU.

## Methods

### Design and setting

The data for this analysis were collected at Lovisenberg Diaconal Hospital in Oslo, Norway as part of a larger cross-sectional study, ‘Safety in Hospital’, conducted on 10 pre-selected screening days between September 2012 and May 2014. The larger study involved conducting standardized risk assessments and assessing the prevalence of falls, pressure ulcers, malnutrition, pain, and other symptoms and comorbidities among inpatients hospitalized in the medical and surgical wards. The hospital’s medical department treats approximately 7800 patients per year, with pulmonary, cardiovascular, gastro-intestinal and infectious diseases being the main disease groups for which medical patients are treated. The surgical department performs elective surgery and about 3000 surgical inpatients are treated annually, including approximately 90 shoulder, 670 hip and 520 knee arthroplasty replacements, and 1700 minor orthopedic, ear/nose/throat and other general operations.

### Study population

All adult inpatients (≥18 years) admitted to one of the hospital’s three medical or two surgical wards by 7 AM on the 10 pre-scheduled screening days (four during the first project year and six during the second) were asked to participate in the study. Patients admitted to Hospice or the intensive care unit or who were cognitively impaired or unable to read Norwegian were not included. Cognitive impairment was determined based on diagnostic information from the medical record and on the clinical judgement of the patient’s primary nurse. Participating patients needed to be able to understand and respond to verbal or written questions. For patients screened on more than one screening day, only data from the date they first consented was included in the analysis. In Year 1 of the study (screening days 1–4), only patients who provided informed consent were screened and included in the study. However, in Year 2 of the study (screening days 5–10), the hospital implemented routine screening as part of standard clinical procedures, and, thus, anonymous screenings of patients who did not consent to the study were included in the analysis as part of the hospital’s quality assurance register.

### Data collection

Second-year nursing bachelor students and ward nurses trained in standardized screening, rigor in research and research ethics performed the initial nutritional screenings and assessed patients for PUs. A registered clinical dietician performed the final nutritional screenings when indicated by the initial screening. Data on age and sex were collected from the patients’ medical records. Body mass index (BMI) was calculated based on height and weight obtained through the nutritional screening or from the medical record. All data were collected on the 10 screening days (Tuesdays and Wednesdays), which were selected based on the availability of the research team.

### Measures

#### Nutritional risk screening

An adapted version of the Nutrition Risk Screening 2002 (NRS 2002) [19] was used to screen for nutritional risk. While the original version uses a BMI cutoff of <20.5, the adapted version uses <20 kg/m^2^, the general international consensus for underweight [18,29,30]. The NRS 2002 screening tool consists of two parts: initial screening to be performed on all patients and final screening to be performed when indicated by the initial screening.

(1) Initial screening:

In this study, the following three initial questions were answered ‘Yes’ or ‘No’ by the patient, their family member or nurse. If all questions were answered ‘No’, the patient was considered to be at low nutritional risk (‘Low risk’) and weekly re-screening was recommended. If one or more questions were answered ‘Yes’, the patient was referred to the registered clinical dietician for final screening.

- Is BMI< 20 kg/m^2^? (Later referred to as ‘BMI < 20’.)
- Has the patient lost weight within the last 3 months? (Later referred to as ‘Weight loss past 3 months’.)
- Has the patient had reduced dietary intake in the last week? (Later referred to as ‘Ate less past week’.)

The NRS 2002 initial screening also includes an item about whether the patient is severely ill (i.e. intensive care patient). However, this item was not applicable for this study because the hospital does not treat severely ill patients with burns, with trauma or in need of transplantation. Patients with severe infection or other serious illness are hospitalized in the intensive care unit and were excluded from this study.

(2) Final screening:

The following factors were evaluated on a 0–3 scale, with 0 indicating ‘low risk’ and 3 ‘high risk’. A total score was determined by summing the two factor scores, and patients 70 years and older had an additional point added to their total score. Patients with a total score of 3 or more (out of maximum score of 7) were considered to be at risk of malnutrition.

- nutritional status (based on BMI category, degree of decrease in dietary intake and degree of weight loss) and
- severity of disease, based on disease-related increased nutritional requirements. For this study, minor elective surgery was assigned a severity disease score of 1 and major surgery was assigned a score of 2.

#### Skin examinations

**Figure 1.**
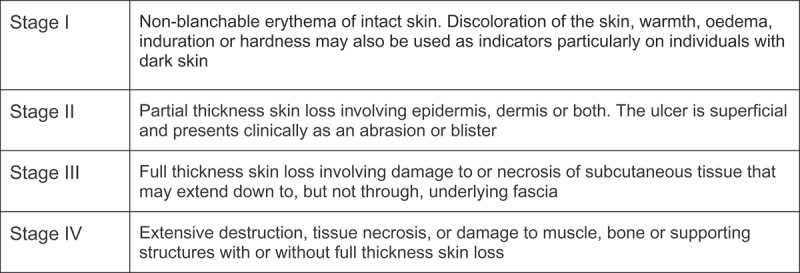
Pressure ulcer classification according to European Pressure Ulcer Advisory Panel [1].

The National Pressure Ulcer Advisory Panel (NPUAP) and EPUAP have defined PUs as localized injury to the skin and/or underlying tissue, usually over a bony prominence, as a result of pressure or pressure in combination with shear [1]. The results of all skin examinations were classified according to the NPUAP/EPUAP classification system, which defines the maximum depth of tissue involvement from Stage I through IV (Figure 1). For the purpose of this study, all abnormal skin exams (Stages I–IV) were considered indicative of PUs.

#### Body mass index

Body mass index was calculated as the patients’ weight in kilograms divided by their squared height in meters. Patients were weighed in the morning, to the nearest 0.1 kilogram, wearing thin clothing, on either a digital portable scale (Soehnle Melody 2.0) or a wheelchair scale (Vetek TI-1200). All scales were calibrated prior to each screening day. A portable digital scale (Seca Alpha Model 770) was used as the ‘gold standard’ for calibration. Height was measured standing (Kawe height measure Model 94112) or in a supine position on a flat bed, read to the closest 0.5 cm and converted to meters. When height or weight could not be measured and if the patients provided consent, the most recent values were obtained from the patients’ medical record and were used in all data analyses.

#### Socio-demographic characteristics

Data on age and sex were retrieved from the patients’ medical record or the quality assurance register using Qlikview software (Qlik Technologies, Inc., Radnor, PA).

### Statistics

Completed screenings were scanned into a research database. SPSS version 22.0 (IBM Corp, Armonk, NY) was used for all statistical analyses. Descriptive statistics (frequencies and means with standard deviations) were used to summarize sample characteristics. Distributions for each variable were assessed to ensure that they met the assumptions of the statistical analyses performed. Independent sample t-tests were used for group comparisons of continuous variables, and separate-variance t-tests were used when the groups had unequal variances. Chi-square tests were used for group comparisons of categorical variables, and Fisher’s Exact Test was used when any of the expected cell frequencies was < 5. Logistic regression was used to determine the unique relationships between initial and final nutrition screening and PUs (i.e. Stages I–IV skin exams), while controlling for the confounding effects of demographic and other clinical factors. Sex and age group were included in all multivariate models to control for any confounding effects they may have. A significance level of 0.05 was used for all analyses. A sample size of 650 was determined to have 80% power to detect an odds ratio of 2.35, based on the hospital’s estimates of 5% pressure ulcer prevalence and 40% malnutrition prevalence (based on NRS 2002).

### Ethics

The study was approved by the Regional Ethical Committee for medical and health-related research ethics (REK South-East) and the hospital management (Reference # 2012/980A). Study participants provided written informed consent to the risk screening and the retrieval of routinely collected clinical data from their medical records. During the second year of the study (the last six screening days), the hospital implemented routine risk screening as part of standard procedures and anonymized data for patients who did not consent were available for analysis through the hospital’s quality assurance register. REK South-East and the Ombudsman at Oslo University Hospital were notified and acknowledged use of the anonymized quality assurance data.

## Results

### Sample characteristics

**Figure 2.**
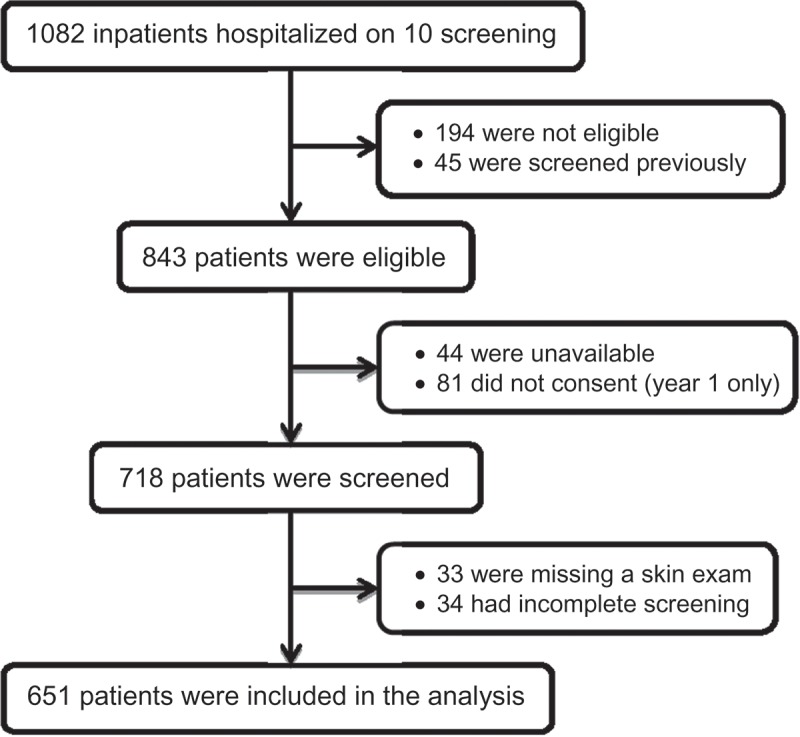
Flow chart of study sample.

Of the 1082 patients in hospital on the 10 screening days, 194 were not eligible (e.g. unable to read Norwegian, cognitively impaired, or under 18 years of age) and 45 were excluded because they had been screened previously. Of the remaining 843 eligible patients, 81 did not consent and 44 were unavailable due to early discharge, operation, or other examination. Of the 718 patients included, 67 were excluded from this analysis due to incomplete initial or final screening (n = 16), missing BMI (n = 18) or missing skin examination (n = 33). For two (< 1%) patients, BMI was obtained from their medical record rather than from study measurements. The final sample included 651 patients (77% of the eligible patients), with complete initial and final screening data and skin examinations (Figure 2).

Sample characteristics for the 651 patients included in the analyses are summarized in Table 1. A comparison of the 651 included and 67 excluded patients indicated that the excluded patients were more likely to be hospitalized on a medical ward (76 vs 55%, p = 0.001). In addition, the 51 excluded patients with complete NRS screening were more likely to be identified as ‘Possible risk’ by the initial screening (70 vs 52%, p = 0.014) and as ‘At risk’ by the final screening (59 vs 34%, p = 0.001). The 34 excluded patients with a complete skin exam were more than twice as likely as included patients to have a PU (18 vs 8%), but this difference was not statistically significant (p = 0.108). There were no age or gender differences between the excluded and included patients.

**Table 1.** Sample characteristics by initial and final nutritional screening status.

		Initial Nutritional Screening			Final Nutritional Screening		
	Total(n = 651)	Low Risk	Possible Risk^a^	Statistics	Low Risk^b^	At Risk	Statistics
		(n = 312)	(n = 339)		(n = 431)	(n = 220)	
Sex, n (%)							
Male	310 (47.6)	168 (54.2)	142 (45.8)	**X^2^[1] = 9.31,**	224 (72.3)	86 (27.7)	**X^2^[1] = 9.69,**
Female	341 (52.4)	144 (42.2)	197 (57.8)	**p = 0.002**	207 (60.7)	134 (39.3)	**p = 0.002**
Age, years							
Mean (SD)	62.9 (17.3)	63.0 (15.6)	62.7 (18.7)	t(643) = 0.22,^c^	61.1 (15.9)	66.3 (19.3)	**t(375) = 3.42**,^c^
Range	19 – 100	20 – 99	19 – 100	p = 0.824	19 – 99	20 – 100	**p = 0.001**
Category, n (%)							
<70 years	416 (63.9)	206 (49.5)	210 (50.5)	X^2^[1] = 1.17,	307 (73.8)	109 (26.2)	**X^2^[1] = 29.7,**
≥70 years	235 (36.1)	106 (45.1)	129 (54.9)	p = 0.279	124 (52.8)	111 (47.2)	**p < 0.001**
Body mass index							
Mean (SD)	25.8 (5.5)	27.0 (4.6)	24.7 (6.0)	**t(628) = 5.46**,^c^	27.2 (5.1)	22.9 (5.2)	**t(649) = 10.3,**
Range	13.6 – 56.6	20.0 – 46.4	13.6 – 56.6	**p < 0.001**	19.6 – 56.6	13.6 – 38.2	**p < 0.001**
Category n (%)				**X^2^[2] = 83.9,**			**X^2^[2] = 165.8,**
<18.5	49 (7.5)	0 (0)	49 (100)	**p < 0.001**	0 (0)	49 (100)	**p < 0.001**
18.5–19.9	31 (4.8)	0 (0)	31 (100)		2 (6.5)	29 (93.5)	
≥20	571 (87.7)	312 (54.6)	259 (45.4)		429 (75.1)	142 (24.9)	
Hospital department				**X^2^[1] = 33.3,**			**X^2^[1] = 40.1,**
Surgical n (%)	293 (45.0)	177 (60.4)	116 (39.6)	**p < 0.001**	232 (79.2)	61 (20.8)	**p < 0.001**
Medical n (%)	358 (55.0)	135 (37.7)	223 (62.3)		199 (55.6)	159 (44.4)	

Percentages are totalled across rows to facilitate comparison of prevalence of nutritional risk in each category of patient characteristics. Statistically significant differences appear in bold.

^a^ Patients identified as having ‘Possible risk’ of malnutrition on initial screening were referred for final screening.

^b^ Includes the 313 patients identified as ‘Low risk’ of malnutrition in the initial screening.

^c^ Separate variance t-test with adjusted degrees of freedom due to unequal variances.

### Initial nutrition screening

Of the 651 patients who received the initial screening, 48% were found to be at low risk of malnutrition (‘Low risk’). The remaining 52% were identified as ‘Possible risk’ on the initial screening based on at least one ‘Yes’ response and were referred for the final screening (Table 1).

Patients identified as being at ‘Possible risk’ were more likely to be female, have BMI < 20, and be hospitalized in the medical department. Women were more likely than men to be at ‘Possible risk’ (57.% vs 45.8%, p = 0.002). By definition, 100% of the patients with BMI < 20 were found to be at ‘Possible risk’, as were 45.4% of patients with BMI ≥ 20. Comparing medical and elective surgical patients, medical patients were more likely to be found at ‘Possible risk’ (62.3% vs 39.6%, p = 0.001). Initial screening status did not differ by age group.

### Final nutrition screening

**Figure 3.**
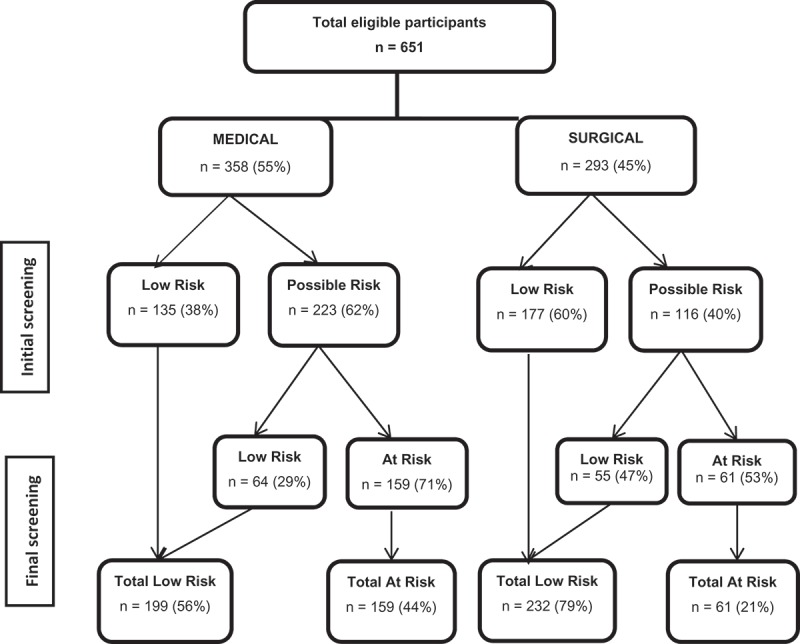
Distribution of nutritional risk using NRS 2002 initial and final screening.

Of the 651 patients included in the final sample, 52% (62.3% of medical patients and 39.6% of surgical patients) were referred for the final screening (Table 1). A total of 33.8% were found to be ‘At risk’ for malnutrition, as determined by the final screening. Women were more likely than men to be found at risk of malnutrition (39.3% vs 27.7%, p = 0.002). Final screening status also differed significantly by age group. For patients 70 years or older, 47.2% were found to be ‘At risk’ for malnutrition, while only 26.2% of patients younger than 70 years were found to be ‘At risk’, p = 0.001). Nearly all (97.5%) of the patients with BMI ˂ 20 and 24.8% with BMI ≥ 20 were determined to be ‘At risk’. The medical patients were more likely to be found ‘At risk’ compared to the elective surgical patients (44.4% vs 20.8%, p < 0.001) (Table 1). Figure 3 summarizes the results from the initial and the final screening for medical and surgical departments.

### Skin examination

Normal skin condition was observed in 597 (91.7%) patients, while 54 (8.3%) had PUs, using the EPUAP/NPUAP classification system (Figure 1). Stage I PU was found in 29 patients (4.5%), while Stage II was observed in 17 (2.6%), Stage III in five (0.8%) and Stage IV in three patients (0.5%). As shown in Table 2, factors associated with prevalence of PUs included age ≥ 70 years (15.7% vs 4.1%, p < 0.001), hospitalized in the medical department (11.7% vs 4.1%, p < 0.001) and BMI ˂ 20 (19.8% vs 6.7%, p < 0.001), where patients with BMI below 18.5 had the highest prevalence of PUs (26.5%). Overweight (BMI 25–29.9) and obese patients (BMI ≥ 30) had similar rates of PU as patients with normal BMI 20–24.9 (3.7% vs 8.9% vs 8.2%, respectively), and were grouped together for subsequent analyses. There was no significant gender difference in PU prevalence.

**Table 2.** Skin examination results in relation to demographic, clinical, and nutritional factors.

		Skin examination		
		Normal	PU Stages I–IV	
	Total	(n = 597)	(n = 54)	
	(n = 651)	(91.7%)	(8.3%)	Statistics
**Demographic vVariables**				
Sex, n (%)				
Male	310 (47.6)	284 (91.6)	26 (8.4)	X^2^[1] = 0.01, p = 0.935
Female	341 (52.4)	313 (91.8)	28 (8.2)	
Age, years				**t(650) = 5.62,**
Mean (SD)	62.8 (17.4)	61.7 (17.2)	75.2 (14.8)	**p < 0.001**
Range	18–100	18–100	30–99	
Category, n (%)				**X^2^[1] = 26.8, p < 0.001**
<70 years	416 (63.9)	399 (95.9)	17 (4.1)	
≥70 years	235 (36.1)	198 (84.3)	37 (15.7)	
**Clinical variables**				
Body mass index (BMI)				t(59.5) = 1.99,
Mean (SD)	25.5 (5.5)	25.7 (5.4)	23.9 (6.3)	p = 0.052^a^
Range	13.6 – 56.6	14.0 – 56.6	13.6 – 42.7	
Category, n (%)				**Fisher’s**
< 18.5	49 (7.5)	36 (73.5)	13 (26.5)	**Exact = 17.4,**
18.5–19.9	31 (4.8)	28 (90.3)	3 (9.7)	**p < 0.001**
≥ 20	571 (87.7)	533 (93.3)	38 (6.7)	
Hospital department, n (%)				**X^2^[1] = 12.4, p < 0.001**
Surgical	293 (45.0)	281 (95.9)	12 (4.1)	
Medical	358 (55.0)	316 (88.3)	42 (11.7)	
**Nutritional dcreening**				
Initial screening, n (%)				**X^2^[1] = 15.6, p < 0.001**
Low risk of malnutrition	312 (47.9)	300 (96.2)	12 (3.8)	Sensitivity: 78%
Possible risk of malnutrition	339 (52.1)	297 (87.6)	42 (12.4)	Specificity: 50%
Final screening, n (%)				**X^2^[1] = 28.4, p < 0.001**
Low risk of malnutrition^b^	431 (66.2)	413 (95.8)	18 (4.2)	Sensitivity: 67%
At risk of malnutrition	220 (33.8)	184 (83.6)	36 (16.4)	Specificity: 69%
Initial screening items, n (%)				
Body mass index				**X^2^[1] = 16.0, p < 0.001**
< 20	81 (12.4)	65 (80.2)	16 (19.8)	
≥ 20	570 (87.6)	532 (93.3)	38 (6.7)	
Weight loss past 3 months?				**X^2^[1] = 4.99, p = 0.026**
Yes	191 (29.3)	168 (88.0)	23 (12.0)	
No	460 (70.7)	429 (93.3)	31 (6.7)	
Ate less past week?				**X^2^[1] = 8.84, p = 0.003**
Yes	240 (36.9)	210 (87.5)	30 (12.5)	
No	411 (63.1)	387 (94.2)	24 (5.8)	

Note. Percentages are totalled across rows to facilitate comparison of prevalence of pressure ulcer in each category. Statistically significant differences appear in bold.

^a^ Separate variance t-test with adjusted degrees of freedom due to unequal variances.

^b^ Includes the 312 patients identified as low risk of malnutrition in the initial screening.

### Associations between nutrition risk and pressure ulcers

Patients identified as being at nutritional risk, either on the initial (‘Possible risk’) or final screening (‘At risk’), were more likely to have a PU (OR = 2.58 and 2.55, respectively) than patients at ‘Low risk’ of malnutrition based on the same screening (initial or final). In addition, each of the three initial nutrition screening items was significantly associated with the skin examination results, with ‘BMI ˂ 20’ (p < 0.001) and ‘Ate less past week’ (p = 0.003) having the strongest associations with PUs (Table 2). Patients with BMI ˂ 20 had nearly three times higher prevalence of PUs compared to patients with BMI ≥ 20 (19.8% vs 6.7%, p ˂ 0.001). Having eaten less in the past week more than doubled the prevalence of PUs (12.5% vs 5.8%, p < 0.003), while weight loss the in past 3 months almost doubled PU prevalence (12.0% vs 6.7%, p = 0.026). As shown in Table 2, the initial screening was more sensitive, but less specific, to the presence of PUs than the final screening.

### Multivariate models

Multivariate models were used to evaluate the usefulness of the initial and final nutritional screening for identifying patients with PUs, while controlling for demographic and other clinical characteristics. Given the differences between medical and surgical patients with respect to risk of malnutrition and PU prevalence, hospital department was also included as a covariate. As shown in Table 3, nutritional risk as determined by the initial screening was a significant predictor of PU (p = 0.011), even after controlling for sex, age, hospital department and BMI. Similar findings were observed for the final nutritional screening (p = 0.008).

**Table 3.** Multivariate Analysis Predicting Pressure Ulcer with Initial or Final NRS 2002 Screening (n = 651).

Model	Variables	Odds rRatio	95% CI	P	Overall model
1	INITIAL NUTRITION SCREENING				X^2^[6] = 55.2, p < 0.001
	Covariates				
	Male sex (ref.: female)	1.361	0.732, 2.532	0.329	
	Age ≥ 70 (ref.: <70)	4.541	2.430, 8.486	**<0.001**	
	Medical patient (ref.: surgical)	2.046	1.001, 4.182	0.050	
	BMI (ref.: ≥ 20)			0.051	
	< 18.5	2.714	1.206, 6.108	**0.016**	
	18.5–19.9	1.072	0.288, 3.989	0.918	
	At nutritional risk based on *initial* screening (ref low nutritional risk)	2.578	1.243, 5.346	**0.011**	
2	FINAL NUTRITION SCREENING				X^2^[6] = 55.2, p < 0.001
	Covariates				
	Male sex (ref.: female)	1.337	0.718, 2.489	0.358	
	Age ≥ 70 (ref.: < 70)	3.932	2.086, 7.410	**<0.001**	
	Medical patient (ref.: surgical)	2.057	1.003, 4.218	**0.049**	
	BMI (ref: ≥ 20)			0.132	
	< 18.5	2.303	0.989, 5.364	0.053	
	18.5–19.9	0.937	0.247, 3.549	0.923	
	At nutritional risk based on *final* screening (ref.: low nutritional risk)	2.552	1.271, 5.125	**0.008**	

ref = reference group

To determine which of the three initial nutrition screening items were most strongly associated with PU when controlling for demographic and other clinical factors, they were evaluated in two multivariate models (Table 4). Table 4 presents a multivariate analysis predicting PUs from the initial screening items, ‘BMI ˂ 20’, ‘Ate less past week’ and ‘Weight loss last 3 months’. In Model 1, all three initial screening items were included and both BMI < 20 (p = 0.006) and ‘Ate less past week’ (p = 0.046) were significantly associated with PUs.

**Table 4.** Multivariate Analysis Predicting Pressure Ulcer from Initial NRS 2002 Screening Items (n = 651).

Model	Variables	Odds ratio	95% CI	P	Overall model
1	ALL 3 INITIAL SCREENING ITEMS				
	Covariates				X^2^[6] = 50.5, p < 0.001
	Male sex (ref.: female)	1.411	0.759, 2.621	0.276	
	Age ≥ 70 (ref.: < 70)	4.519	2.428, 8.413	**<0.001**	
	Medical patient (ref.: surgical)	2.197	1.072, 4.502	**0.032**	
	Initial Nutritional Screening Items				
	BMI < 20 (ref.: ≥ 20)	2.726	1.328, 5.593	**0.006**	
	Weight loss in last 3 months (ref.: no weight loss)	1.029	0.530, 1.997	0.933	
	Ate less in past week	1.906	1.011, 3.592	**0.046**	
	(ref.: ate normally)				
2	COMBINED SCREENING ITEMS				
	Covariates				X^2^[5] = 55.7, p < 0.001
	Male sex (ref.: female)	1.424	0.767, 2.646	0.263	
	Age ≥ 70 (ref.: < 70)	4.551	2.437, 8.496	**<0.001**	
	Medical patient (ref: surgical)	2.091	1.026, 4.262	**0.042**	
	Initial Nutritional Screening Items				
	BMI <.20 (ref.: ≥ 20)	2.510	1.230, 5.123	**0.011**	
	Weight loss OR ate less (ref.: no weight loss and ate normally)	2.747	1.417, 5.326	**0.003**	

ref = reference group

Given the correlation between weight loss in the past 3 months and eating less in the past week (r = .33, p < 0.001), these items were combined into a composite item which was included with BMI < 20 in Model 2. Using this approach, it was determined that patients who had eaten less in the past week or had lost weight in the past 3 months had significantly greater risk of PUs than patients who had neither (OR 2.74, p = 0.003), even when controlling for the known risk factors of older age, hospitalization in the medical department and BMI < 20. The combined item was an even stronger predictor of PUs than BMI < 20.

## Discussion

To our knowledge, this study is the first to show the significant association between the NRS 2002 and PUs in a mixed hospital population. Recently published Norwegian data indicate that the NRS 2002 initial screening is strongly associated with hospitalization, morbidity, poor outcome and mortality [26]. In addition, other nutritional assessments, including the SGA (Subjective Global Assessment), MNA (Mini Nutritional Assessment), and MUST (Malnutrition Universal Screening Tool) have been associated with PUs in elderly and hospital populations in recent studies [7,12,31]. Malnourished patients (determined by SGA) had a higher prevalence of PU than adequately nourished patients [12]. For older patients, with an average age of 85 years, MNA score > 8 was found to be more sensitive than SGA in detecting PU development [31]. Using MUST in a hospital setting, older age, BMI < 18.5, reduced food intake in the past week and unintentional weight loss in the past 3 months were strongly related to manifestation of PUs [7].

In this study, both the initial and the final NRS 2002 screenings were significantly associated with the presence of PUs, even after controlling for age, sex, BMI and hospital department. For hospital inpatients, it is likely that these risk factors are known at admission. Thus, adding nutritional risk screening may be a useful indicator of PUs beyond the already known risk factors.

It is important to note that most (85%) of the identified PUs were at an early stage of development (Stage I or II). Thus, risk of malnutrition as assessed by NRS 2002 may have the potential to help with the early identification of patients at risk of developing more serious PUs in the future. Longitudinal studies evaluating the use of the NRS 2002 for predicting future development of PUs are warranted based on the cross-sectional findings of this study.

Although a significant association was found between PUs and the NRS 2002 final screening, the initial screening is less time- and resource-consuming, thereby allowing for more rapid and targeted assessment and treatment. The initial screening was more sensitive, but less specific to the presence of PUs than the final screening. The disadvantage of using the less specific initial screening as an indicator of PUs is that many of the patients identified as being at possible risk of malnutrition will not have and may not develop PUs. Nonetheless, given the negative impact of PUs on both patients and healthcare costs, it would likely be an acceptable trade-off to initiate PU preventive procedures for some patients who may not need them rather than fail to provide such preventive measures to some of those who do. Moreover, the initial NRS 2002 screening does identify nearly half of all patients as being at low risk of malnutrition, which may help target PU preventive procedures to those who most need them.

In the multivariate analysis predicting the presence of PUs from the three initial nutrition screening items (Table 4, Model 1), ‘BMI < 20’ and ‘Ate less past week’ were significantly associated with PU, but weight loss was not. This finding might be due to multicollinearity among the initial screening items, which can result in reduced significance when the correlated items are included in the same model. However, a combination of the eating less and weight loss items (Table 4, Model 2) was significantly associated with PUs, even after controlling for the effects of older age, being a medical patient and having a BMI < 20. The combination of these two items might be helpful for identifying patients with increased risk of PUs due to malnutrition, regardless of the patient’s BMI and other known risk factors.

A closer look at the individual initial screening items might give guidance to their significance. Low BMI has been consistently identified as having a negative impact on health outcomes [30]. BMI ˂ 18.5 is established by ESPEN guidelines as one diagnostic criterion for malnutrition [30]. Using NRS 2002, this criterion places all patients with BMI ˂ 18.5 in the ‘At risk’ group, as determined by the final screening. BMI ˂ 18.5 was also found to be strongly associated with PUs in a prior study [7]. In our study, patients with BMI ˂ 20 had a significantly higher incidence of PUs than patients with BMI ≥ 20, and those with BMI < 18.5 had a particularly high incidence of PUs (26.5%). Low BMI was consistently and strongly associated with the presence of PUs in the current analyses and thus requires close attention in all healthcare services.

‘Weight loss in the last 3 months’ occurred in almost half (43%) of the patients with PUs in this study. Weight loss causes the body to go into a catabolic state, which has a negative impact on the healing process [32]. Disease-related weight loss is common, as about 70% of hospital patients are discharged with a lower body weight than at admission [18]. Thus, healthcare professionals are strongly encouraged to limit in-hospital weight loss due to its negative impacts on health outcomes, such as poor healing, additional infections, malnutrition and longer hospitalization [18]. Weight loss is recommended for some obese patients prior to elective orthopedic surgery. However, to reduce the risk of poor wound healing and PUs, it might be suggested that patients cease weight loss before surgery to stimulate a preoperative anabolic state [33].

Eating less in the past week was reported among 56% of patients with PUs. This indicates that the patients were most likely in a catabolic state when screened, a common situation when admitted to hospital. Insufficient dietary intake has been shown to be inversely related to patient recovery [18,33] and should be addressed for immediate proper nutritional care. A decline in nutritional status will most likely hamper the healing process and increase length of hospital stay. Longer hospitalization due to PUs and/or malnutrition has an indisputable negative impact on the patient and ward personnel, as well as local and national healthcare budgets [3,7,12,23,24].

NRS 2002 is a screening tool intended to detect patients who might benefit from nutritional support [19], where the initial screening can be used to quickly detect patients at possible risk of malnutrition and the final screening further identifies those at risk of malnutrition based on a more thorough evaluation of the patient’s nutritional risk factors. The intention of PU risk assessment and classification is to detect patients at risk of developing PUs and classify the maximum depth of existing PUs [1]. National efforts aimed at reducing PU risks and providing proper treatment do not yet seem to have resulted in significantly lower PU prevalence [10,11]. PU screening and risk assessment procedures are often not conducted as recommended, which indicates that there are still issues to address to achieve optimal compliance and patient safety results. The significant association between the initial NRS 2002 screening and PUs suggests that it could be an easier, less time-consuming and efficient method of detecting patients and may even have the potential to identify patients at risk of developing PUs in the future.

### Strengths and limitations

A strength of this study is its focus on the general medical and elective surgery hospital population and exclusion of ICU patients. While heightened risk of malnutrition and PU development has been well-documented among ICU patients, the association between risk of malnutrition and the presence of PUs in the general medical and elective surgery hospital population has not been well-documented.

The most significant limitation of this study is the cross-sectional study design, which precluded determination of whether the NRS 2002 is useful for identifying patients at risk for the development of future PUs. However, based on the current findings, additional longitudinal studies of the relationship between the NRS 2002 and PU risk are warranted. In addition, the eligibility criteria used in this study likely resulted in the exclusion of patients at highest risk of PUs (e.g. those with cognitive impairment), and, thus, the results may underestimate the associations between risk of malnutrition and the likelihood of PUs in the general hospital population. Furthermore, screenings were conducted on Tuesdays and Wednesdays only, and it cannot be ruled out that this limited selection of screening days caused systematic bias in the data collected. Finally, this study did not include other important risk factors for PUs, such as functional capacity and prior hospitalizations, and did not include any clinical outcomes regarding the effect of nutritional and/or PU treatment.

### Relevance to clinical practice

Nurses are close to the patients at all times, and are thus important contributors to systematic nutritional risk and PU screening, assessment, documentation and monitoring. Simplified methods for screening would most likely be welcomed in an otherwise loaded ward schedule. Targeted and proper education is crucial for optimal management of screening methods. Registered clinical dieticians represent an important group in training and facilitating individual nutritional treatment.

## Conclusion

To our knowledge this cross-sectional study is the first to document an association between nutritional risk screening using the NRS 2002 and the presence of PUs in a general inpatient population. The results indicated that both the initial and final screenings were significantly associated with PUs. Further research is needed to determine whether the NRS 2002 is useful in predicting which patients will develop PU in the future. Rapid identification and targeted nutritional and PU treatment will likely have significant benefits for patient outcomes, healthcare organizations and the healthcare economy.

## References

[1] European Pressure Ulcer Advisory P Prevention and treatment of pressure ulcers : clinical practice guideline. Washington (DC): National Pressure Ulcer Advisory Panel; 2014.

[2] Hengstermann S, Fischer A, Steinhagen-Thiessen E, et al Nutrition status and pressure ulcer: what we need for nutrition screening. JPEN J Parenter Enteral Nutr. 2007;31(4):288–11. 17595437 10.1177/0148607107031004288

[3] Litchford MD, Dorner B, Posthauer ME. Malnutrition as a precursor of pressure ulcers. Adv Wound Care. 2014;3(1):54–63. 10.1089/wound.2012.0385 PMC3899999 24761345

[4] Little MO Nutrition and skin ulcers. Curr Opin Clin Nutr Metab Care. 2013;16(1):39–49. 23222706 10.1097/MCO.0b013e32835bc0a1

[5] Berlowitz DR Prevention of pressure ulcers. UpToDate. Walter and Kluwer; 2014.

[6] Hisashige A, Ohura T Cost-effectiveness of nutritional intervention on healing of pressure ulcers. Clin Nutr. 2012;31(6):868–874. 22626888 10.1016/j.clnu.2012.04.013

[7] Tsaousi G, Stavrou G, Ioannidis A, et al Pressure ulcers and malnutrition: results from a snapshot sampling in a university hospital. Med Princ Pract. 2015;24(1):11–16. 25402507 10.1159/000368360 PMC5588197

[8] Vanderwee K, Clark M, Dealey C, et al Pressure ulcer prevalence in Europe: a pilot study. J Eval Clin Pract. 2007;13(2):227–235. 17378869 10.1111/j.1365-2753.2006.00684.x

[9] Jiang Q, Li X, Qu X, et al The incidence, risk factors and characteristics of pressure ulcers in hospitalized patients in China. Int J Clin Exp Pathol. 2014;7(5):2587–2594. 24966973 PMC4069923

[10] Bredesen IM, Bjøro K, Gunningberg L, et al Patient and organisational variables associated with pressure ulcer prevalence in hospital settings: A multilevel analysis. BMJ Open. 2015;5:8. 10.1136/bmjopen-2015-007584 PMC4554903 26316647

[11] Bjøro K, Ribu L Pilotstudie av trykksårprevalens i et norsk sykehus. Sykepleien Forskning. 2009;4:298–305.

[12] Brito PA, de Vasconcelos Generoso S, Correia MI Prevalence of pressure ulcers in hospitals in Brazil and association with nutritional status–a multicenter, cross-sectional study. Nutrition. 2013;29(4):646–649. 23466049 10.1016/j.nut.2012.11.008

[13] Stechmiller JK, Cowan L, Whitney JD, et al Guidelines for the prevention of pressure ulcers. Wound Repair Regen. 2008;16(2):151–168. 18318801 10.1111/j.1524-475X.2008.00356.x

[14] Bennett G, Dealey C, Posnett J The cost of pressure ulcers in the UK. Age Ageing. 2004;33(3):230–235. 15082426 10.1093/ageing/afh086

[15] Silva AJ, Pereira SM, Rodrigues A, et al Economic cost of treating pressure ulcers: a theoretical approach. Rev Esc Enferm USP. 2013;47(4):971–976. 24310698 10.1590/S0080-623420130000400028

[16] Braut GS Lecture: trykksår i eit samfunnsperspektiv - spørsmål frå ein samfunnsmedisinar? 14(February):2008 Available from: http://www.nifs-saar.no/pdf/2008/2008_geir_sverre_braut.pdf

[17] Severens JL, Habraken JM, Duivenvoorden S, et al The cost of illness of pressure ulcers in The Netherlands. Adv Skin Wound Care. 2002;15(2):72–77. 11984050 10.1097/00129334-200203000-00008

[18] Stratton RJ, Green CJ, Elia M Disease-related malnutrition : an evidence-based approach to treatment. Wallingford: CABI; 2003.

[19] Kondrup J, Allison SP, Elia M, et al ESPEN Guidelines for Nutrition Screening 2002. Clin Nutr. 2003;22(4):415–421. 12880610 10.1016/s0261-5614(03)00098-0

[20] Guttormsen AB Helsedirektoratet Avdeling e. Nasjonale faglige retningslinjer for forebygging og behandling av underernæring. Oslo: Helsedirektoratet, Avdeling ernæring; 2009.

[21] Rasmussen HH, Holst M, Kondrup J Measuring nutritional risk in hospitals. Clin Epidemiol. 2010;2:209–216. 21042553 10.2147/CLEP.S11265 PMC2964075

[22] Hiesmayer Decreased food intake is a risk factor for mortality in hospitalised patients: the NutritionDay survey 2006. Clin Nutr. 2009;28:484–491. 19573957 10.1016/j.clnu.2009.05.013

[23] Lim SL, Ong KCB, Chan YH, et al Malnutrition and its impact on cost of hospitalization, length of stay, readmission and 3-year mortality. Clin Nutr. 2011;31(3):345–350. 22122869 10.1016/j.clnu.2011.11.001

[24] Pirlich M, Schütz T, Norman K, et al The German hospital malnutrition study. Clin Nutr. 2006;25(4):563–572. 16698132 10.1016/j.clnu.2006.03.005

[25] Ray S, Laur C, Golubic R Malnutrition in healthcare institutions: A review of the prevalence of under-nutrition in hospitals and care homes since 1994 in England. Clin Nutr. 2014;33(5):829–835. 24238787 10.1016/j.clnu.2013.10.017

[26] Tangvik RJ, Tell GS, Eisman JA, et al The nutritional strategy: four questions predict morbidity, mortality and health care costs. Clin Nutr. 2014;33(4):634–641. 24094814 10.1016/j.clnu.2013.09.008

[27] Tangvik RJ, Tell GS, Guttormsen AB, et al Nutritional risk profile in a university hospital population. Clin Nutr. 2015;34(4):705–711. 25159298 10.1016/j.clnu.2014.08.001

[28] Helsedirektoratet.Pasientsikkerhetsprogrammet-I trygge hender. Forebygging av trykksår.2015. Available from: http://www.pasientsikkerhetsprogrammet.no/no/I+trygge+hender/In+English.

[29] Physical status: the use and interpretation of anthropometry Report of a WHO Expert Committee. World Health Organ Tech Rep Ser. 1995;854:1–452. 8594834

[30] Cederholm T, Bosaeus I, Barazzoni R, et al Diagnostic criteria for malnutrition - An ESPEN Consensus Statement. Clin Nutr. 2015;34(3):335–340. 25799486 10.1016/j.clnu.2015.03.001

[31] Yatabe MS, Taguchi F, Ishida I, et al Mini nutritional assessment as a useful method of predicting the development of pressure ulcers in elderly inpatients. J Am Geriatr Soc. 2013;61(10):1698–1704. 24083424 10.1111/jgs.12455

[32] Molnar JA, Underdown MJ, Clark WA Nutrition and chronic wounds. Adv Wound Care. 2014;3(11):663–681. 10.1089/wound.2014.0530 PMC4217039 25371850

[33] Lui M, Jones CA, Westby MD Effect of non-surgical, non-pharmacological weight loss interventions in patients who are obese prior to hip and knee arthroplasty surgery: a rapid review. (Research)(Clinical report). Syst Rev. 2015;4:121. 26410227 10.1186/s13643-015-0107-2 PMC4584125

